# Influence of body condition score at calving on the metabolic status and production performance of Murrah buffaloes (*Bubalus bubalis*) during the transition period

**DOI:** 10.5713/ajas.17.0223

**Published:** 2017-07-17

**Authors:** Nelson Carvalho Delfino, Lucas Fialho de Aragão Bulcão, Henry Daniel Ruiz Alba, Mauricio Xavier da Silva Oliveira, Filipe Pinheiro Soares de Queiroz, Gleidson Giordano Pinto de Carvalho, Francisco Palma Rennó, José Esler de Freitas

**Affiliations:** 1 Department of Animal Sciences, School of Veterinary Medicine and Animal Science, Federal University of Bahia (UFBA), Salvador, BA, 40.170-110, Brazil; 2 Department of Animal Nutrition and Production, School of Veterinary Medicine and Animal Science, University of São Paulo (USP), Pirassununga, SP 13635-900, Brazil

**Keywords:** Body Condition Score, Lactation, Metabolism, Pregnancy, Buffaloes

## Abstract

**Objective:**

The purpose of this study was to evaluate the influence of body condition score (BCS) at calving on the metabolic status of female Murrah buffaloes in the transition period.

**Methods:**

Thirty-seven pregnant buffaloes (multiparous) were selected and monitored during the transition period based on their body condition score and on the estimated calving date. Two groups were formed: i) buffaloes with a BCS>3.5 (n = 17); this group was classified and named ‘high BCS at calving’ (HBCS); and ii) buffaloes with a BCS≤3.5 (n = 20); this group was classified and named ‘low BCS at calving’ (LBCS). All animals were monitored during the last 30 days of pregnancy and the first 70 days post-calving and kept in the same environment and under the same feeding and management conditions. Mean values for BCS at calving were 2.98±0.9 (mean±standard error of the mean [SEM]) and 4.21±0.9 (mean±SEM) for the HBCS and LBCS groups, respectively.

**Results:**

The HBCS group showed higher milk fat content (p = 0.007) and milk fat yield (p = 0.027) and a higher concentration of milk urea nitrogen (p = 0.001) than LBCS buffaloes, which in turn had a lower urine pH value (p = 0.033) than HBCS buffaloes in the pre-calving period (7.86 for HBCS vs 7.76 for LBCS). The HBCS animals had a higher concentration of erythrocytes (p = 0.001) and hematocrit (p = 0.012) post-calving and a higher hemoglobin concentration (p = 0.004) pre-calving.

**Conclusion:**

Buffaloes during the transition period exhibited some variations in the oxidative stress related to their metabolic status. After calving, buffaloes with a high BCS at calving and greater lipid mobilization have a more marked alteration in oxidative status, but improved production performance.

## INTRODUCTION

The transition period is characterized by a decrease in feed intake and an increase in fetal growth and homeorhetic adaptations [1], including mobilization of body reserves. The difference between the lower nutrient intake and increased energy requirements of females for maintenance, own body and fetal growth, and production of colostrum and milk for the newborn leads to a physiological imbalance called negative energy balance [2,3].

Calorimetry studies on high-producing dairy cows have estimated a negative energy bal ance of −22 MJ/d in the sixth week of lactation, decreasing to −9 MJ/d in the twelfth week [4]. To reduce the intensity of the negative energy balance through homeorhetic adaptations, a number of metabolic reactions are initiated, with a consequent mobilization of body reserves (especially fats) and reduction of body weight (BW). The lipolytic activity observed with the reduction of body reserves could contribute to 33% of milk yield or maintain the production of 120 to 550 kg of milk in the first weeks of production, in dairy cows [5].

The lipolytic activity is correlated with the formation of ke tone bodies, which, when in excess, are negatively correlated with the animal health, causing metabolic disorders (ketosis, hypocalcemia, abomasal displasia, and fatty liver), and a reduction in immune response generated by oxidative stress [2,3].

Oxidative stress is associated with the body condition score (BCS) [6], a method to assess the body condition of the animal that is highly correlated with the mobilization of body reserves. Body condition score has the advantage of being a quick, effective, economic, and non-invasive approach. Despite its subjective nature [7], BCS is very important in the establishment of adequate production-management techniques [5,8].

The objective of this study was thus to evaluate the influ ence of BCS at calving on the metabolic status of female Murrah buffaloes in the transition period. Our hypothesis is that a higher BCS at calving positively influences the concentrations of metabolites in the post-calving transition period, changing the oxidative status and increasing the production performance of these animals.

## MATERIALS AND METHODS

### Ethical aspects and location

This study was conducted in strict conformity with the recommendations of Brazil’s National Council for Animal Experimentation (CONCEA). The undertaken experimental procedures were approved by the Ethics Committee in Animal Use of the School of Veterinary Medicine and Animal Science of the Federal University of Bahia, Bahia State, Brazil (no. 39-2014).

### Animals, housing, and feeding

The experiment was conducted in a commercial dairy herd of Murrah buffaloes (*Bubalus bubalis*). The average milk yield per lactation (300 days in milk) of the herd was higher than 2,400 kg. Thirty-seven pregnant buffaloes (multiparous) were selected based on their BCS and on the estimated calving date. Two groups of BCS at calving were formed, as follows: i) buffaloes with a BCS >3.5 (n = 17); this group was classified and named ‘high BCS at calving’ (HBCS); and ii) buffaloes with a BCS≤3.5 (n = 20); this group was classified and named ‘low BCS at calving’ (LBCS). All animals were monitored during the last 30 days of gestation and the first 70 days post-calving and were kept in the same environment and under the same feeding and management conditions. Mean values for BCS at calving were 2.98±0.9 (mean±standard error of the mean [SEM]) and 4.21±0.9 (mean±SEM) for groups LBCS and HBCS, respectively.

The BCS was statistically different between the groups (p < 0.001). In the post-calving period, all buffaloes were fed chopped elephant grass (*Penissetum purpureum*) and a concentrate in the form of total mixed ration immediately after milking. The diet (Table 1) was formulated to meet the requirements of lactating buffaloes producing 6.0 kg/d milk with 7.0% fat and 4.2% crude protein (CP) according to the recommendations of Paul and Lal [9]. After the morning feeding, all buffaloes post-calving were kept in paddocks with *Brachiaria decumbens* grass (nutrient composition [g/kg dry matter {DM}]: 216.5 DM, 110.1 CP, 727.4 neutral detergent fiber [NDF], and 142.1 non-fibrous carbohydrates [NFC]). The chemical composition of elephant grass can be viewed in the footnote of Table 1.

### Sample collection and chemical analysis

Feed samples were processed through a Wiley mill with 1-mm sieves (AH Thomas, Philadelphia, PA, USA) and analyzed for the DM (AOAC 950.15), ash (AOAC 942.05), ether extract (EE, AOAC 920.39), CP (N×6.25; AOAC 984.13), and lignin (AOAC 973.18) contents according to the methods described by AOAC [10]. The NDF was analyzed using α-amylase without addition of sodium sulfide to the detergent (TE-149 analyzer, Tecnal Equipments for Laboratory Inc., Piracicaba, Brazil). The concentrations of NFC were estimated according to [11], as follows: NFC = 100 − [(% CP − % CP from urea+% urea)+ % EE+% ash+% NDF].

### Milk yield and composition

Buffaloes were milked mechanically daily at 0600 h, and their milk yield was measured by an automatic milk meter (Delaval, Tumba, Sweden). Milk yield was corrected to 4.0% fat (FCM) according to Di Paolo [12]: Y = 1+0.01155 [(X–40)+(Z–31)], where Y is the amount (kg) of FCM equivalent to 1 kg of milk produced and X and Z are the grams of fat and protein present in 1 kg of milk produced, respectively. Milk samples were collected automatically (MM6, DeLaval, Sweden) once weekly (7, 14, 21, 28, 35, 42, 49, 56, 63, and 70 days), according to the milk production of each animal in each milking. Milk samples were analyzed fresh for fat, protein, lactose, milk urea nitrogen (MUN), and somatic cell count following Campanile et al [13].

### Body condition score, body weight, and clinical parameters

During the study, BCS was evaluated using the body-condition scoring method for Murrah buffaloes according to Anitha et al [7]. A graph was used for the classification of condition on a scale of 1 to 5 using 0.5-point subunits. Measurements were taken weekly by two trained evaluators pre-calving (−28, −21, and −7 days), on the calving date, and post-calving (+7, +14, +21, +28, +35, +42, +49, +56, +63, and +70 days). The BW was obtained on the same days and used to determine the changes in body condition score and in body weight (BWC). Rectal temperature and heart rate (beats/min, using a stethoscope) were also measured.

### Analysis of metabolic status indices

Blood samples were drawn weekly pre-calving (−28, −21, and −7 days), on the calving date, and post-calving (+7, +14, +21, and +28 days). Immediately after collection, samples were centrifuged at 2,000×*g* for 15 min at room temperature to obtain the plasma. Plasma samples were sent to the laboratory and analyzed for complete blood count (erythrocytes, hemoglobin, and hematocrit); mean corpuscular hemoglobin concentration (MCHC), by the cyanmethemoglobin colorimetric technique; mean corpuscular volume (MCV), by the micro-hematocrit method; and leukogram (leukocytes, segmented neutrophils, red blood cells, lymphocytes, and neutrophils), by the May-Grunwald Giemsa method. The serum was transferred to plastic tubes that were identified and stored at −20°C until analyses. Analyses were performed using commercial kits in an automatic biochemistry analyzer (BioSystems, Foster City, CA, USA). Blood samples were harvested to measure the serum glucose (K048, Bioclin, Belo Horizonte, Brazil), total protein (K031, Bioclin, Brazil), albumin (K040, Bioclin, Brazil), total cholesterol (K083, Bioclin, Brazil), urea (K056, Bioclin, Brazil), triglycerides (K117, Bioclin, Brazil), calcium (K051, Bioclin, Brazil), and phosphorus (K068, Bioclin, Brazil).

Urine samples were collected from all animals approxi mately four hours after the morning feeding, when the buffaloes urinated spontaneously. Urine aliquots of 10 mL were immediately diluted with 40 mL 0.036 N sulfuric acid and stored at −20°C for later analyses pre-calving (−28, −21, and −7 days), at calving (until 24 h post-calving), and post-calving (+7, +14, +21, +28, +35, +42, +49, +56, +63, and +70 days). The urine pH values were determined using a digital pH meter (MB-10, Marte Científica, MG, Brazil). In these samples, we determined the concentrations of urea (fixed-time kinetic method; K056, Bioclin, Brazil); uric acid (UA; enzymatic colorimetric method - K139, Bioclin, Brasil); calcium (Ca; end point colorimetric method - Arzenazo III - K051, Bioclin, Brazil); and sulfur (S) and chlorine (Cl; mercury thiocyanate colorimetric method - K050, Bioclin, Brazil). Potassium (K) was determined using a MH 9180 ion-selective device (MH LabISE 9180, Belo Horizonte, Brazil).

### Statistical analysis

The data were analyzed by the PROC MIXED procedure of SAS [14], according to the model of repeated measures over time, with normality of residuals and homogeneity of variances checked by the PROC UNIVARIATE procedure. The model was used to estimate the effect of physiological stage (week), BCS group (Group 1: BCS>3.5, or HBCS; Group 2: BCS≤3.5, or LBCS), and their interaction on oxidative-status indices:

$${\text{Y}}_{ijk}=\mu +{\text{W}}_{i}+{\text{BCS}}_{j}+{\left(\text{W}\times \text{BCS}\right)}_{ij}+{\text{e}}_{ijk}$$

Where Y*_ijk_* = dependent variable; μ = overall mean of the population; W*_i_* = mean effect of the physiological stage (weeks) (*i* = pre-calving, post-calving) with the physiological stage as a repeated factor; BCS*_j_* = mean effect of BCS group (j = 1 and 2); and e*_ijk_* = unexplained residual element assumed as independent and normally distributed. For each analyzed variable, the buffaloes from both BCS groups were subjected to three covariance structures: compound symmetry, autoregressive order, and unstructured covariance. The data were analyzed on sampling days relative to the calving date, with day 0 representing the calving date.

## RESULTS

### BCS and clinical parameters

**Figure 1:**
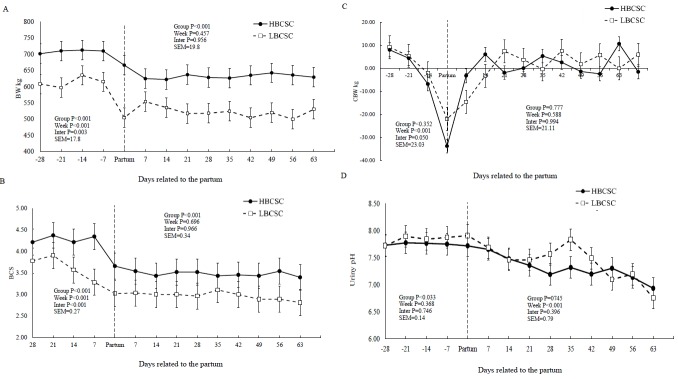
Body weight (BW) (A), body condition score (BCS) (B), change body weight (CBW) (C), and urinary pH values (D), in buffaloes with low body score condition at calving (HBCSC) (Group 1: mean of BCSC = 2.98), and high body score condition at calving (LBCSC) (Group 2: mean of BCSC = 4.21). ^*^ p<0.05, between LBCSC and HBCSC groups.

The HBCS group had a higher average BW and BCS than LBCS group in the pre- (p = 0.001) and post-calving (p = 0.001) periods (Table 2). There was an effect of weeks (p = 0.001) and an interaction effect between weeks and groups for BW (p = 0.001) and BCS (p = 0.001) during the pre-calving period (Figure 1A, 1B). However, there was no difference between the groups for BWC pre- and post-calving. There were week effects (p = 0.001) on BWC pre-calving (Figure 1C). HBCS buffaloes exhibited higher BCS values than the LBCS group during the pre- (p = 0.001) and post-calving (p = 0.001) periods (Table 2).

The HBCS group showed a higher urine pH value (p = 0.033) than the LBCS group pre-calving (7.76 vs 7.86, respectively) (Figure 1D, Table 2). There was an effect of weeks (p = 0.001) for the urine pH and heart rate values (p = 0.029) during the post-calving period.

### Milk yield and composition

**Figure 2:**
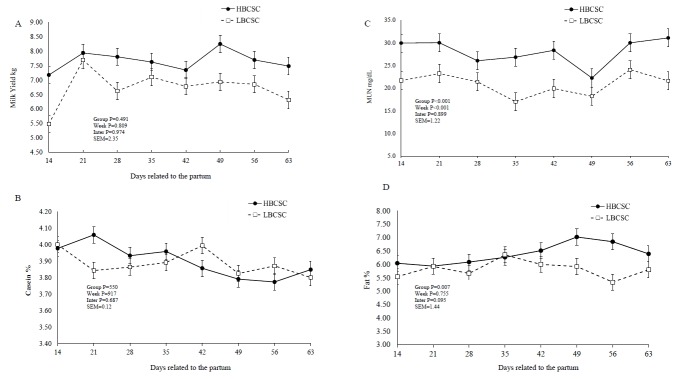
Milk yield (A), casein content (B), milk urea nitrogen (MUN) (C), and milk fat content (D), in buffaloes with low body score condition at calving (HBCSC) (Group 1: mean of BCSC = 2.98), and high body score condition at calving (LBCSC) (Group 2: mean of BCSC = 4.21). ^*^ p<0.05, between LBCSC and HBCSC groups.

There were no differences for milk yield, FCM, total solids, solids nonfat, protein, and lactose between the groups (Table 3). However, the HBCS group had a higher milk fat content (p = 0.007) and milk fat yield (p = 0.027) than the LBCS group (Table 3). There were week effects for the milk protein and lactose concentrations. The HBCS group had a higher MUN content (p = 0.001) than the LBCS buffaloes (28.7 vs 21.2, respectively) (Figure 2C, Table 3). There was a week effect in the lactation period for milk casein concentration, casein as a percentage of crude protein, and MUN (Figure 2B).

### Metabolic parameters

There were no differences in the concentrations of erythrocytes, hematocrit, segmented neutrophils, red blood cells, and lymphocytes between the groups in the pre-calving period (Table 4). In the same way, no differences were detected for the concentrations of MCHC, hemoglobin, red blood cells, and lymphocytes between the BCS groups post-calving. However, the HBCS group exhibited higher concentrations of erythrocytes (p = 0.001) and hematocrit (p = 0.012) post-calving and higher levels of MCHC (p = 0.002) and hemoglobin (p = 0.004) pre-calving (Table 4). The HBCS group showed lower concentrations of MCV (p = 0.001), leukocytes (p = 0.001), and neutrophils (p = 0.032) pre- and post-calving (MCV [p = 0.001], leukocytes [p = 0.016], and neutrophils [p = 0.034]). There was a week effect in the post-calving period for the concentrations of erythrocytes, hematocrit, and leukocytes and in the pre-calving period for hemoglobin and leukocytes.

No differences were observed for the concentrations of glu cose, urea, triglycerides, and phosphorus between the two groups pre- and post-calving (Table 5). The HBCS group presented higher total plasma protein (p = 0.035), albumin (p = 0.036), and globulin (p = 0.046) in the pre-calving period. However, the LBCS group showed a higher total cholesterol (p = 0.029) and calcium (p = 0.003) post-calving. There was an effect of weeks on the concentrations of glucose (p = 0.040) in the pre-calving period and for concentrations of urea (p = 0.004) and total cholesterol (p = 0.005) post-calving.

Urinary concentrations of K, S, Ca, urea, and uric acid did not differ between the two groups during the pre- and post-calving periods (Table 6). Likewise, there was no difference for the Cl concentration between the groups pre-calving. However, the LBCS group showed a higher Cl content (p = 0.010) pre-calving. There was a week effect for the concentrations of urea pre- (p = 0.007) and post-calving (p = 0.014). There was an interaction effect between weeks and BCS groups for the K concentration (p = 0.030) post-calving.

## DISCUSSION

It is known that transition dairy cows with a high BCS lose more BW and body condition than leaner cows [15,16]. Over time, buffalo rearing has shifted from a backyard activity to commercial farms and big companies. The immense popularity of buffalo milk and meat-based products has allowed buffalo production to follow the dairy cattle industry. However, in order for this species to perform optimally under the pressure of intensive production systems, buffalo breeds must be improved and research should be conducted with a clear focus on the transition between different physiological stages and alternations in the lipid metabolism [13,17]. The BCS at calving explains the concept of homeorhesis, which was defined decades ago as a set of metabolic alterations in the animal that has a genetic key to safeguard important biological functions such as the survival of the newborn (through milk provision) or reproduction [18].

During the pre- and post-calving periods, the HBCS group had higher BW and BCS than the LBCS buffaloes. These results are related to the method adopted and the correlation between BW and BCS [7,16]. We observed that the BW loss and the BCS change (65.2 kg and 0.78 points for HBCS vs 51.5 kg and 0.64 points for LBCS) of the groups during the transition found between the pregnant non-lactating and lactating non-pregnant physiological stages (Figure 1C) was mainly due to the increase in BCS before delivery.

The animals from the HBCS group lost 9.6% of their initial BW, while the LBCS animals lost 9.0% of their weight (Table 2) in the pre-calving period. The recommended BCS at calving for dairy cows may be different for dairy buffaloes due to differences in metabolism between the species. The endocrine profiles change [17] and the lipolysis and lipogenesis are regulated to increase the lipid reserves during pregnancy; moreover, the lipid metabolism is regulated by homeostatic and homeorhetic mechanisms [18]. Lacetera et al [19] concluded that, after calving, cows that showed an elevated BCS at calving and high lipid mobilization displayed a more pronounced alteration in oxidative status. These conditions may make the cows more sensitive to oxidative stress. In female beef cattle, blood somatropin concentrations are lower and the insulin concentration is higher when compared with dairy-purpose breeds. Breeds have shown decreased milk production according to their dry matter intake [20]. In this study, we have shown that BW and BCS were lower for the LBCS group, indicating that bubaline females present a similar physiological and metabolic pattern to that of female beef cattle.

We measured some clinical parameters regarding oxidative stress during the change in physiological stage and there was no difference in RT between the groups. Rectal temperature is an important measurement in the physiological assessment; it is correlated with oxidative stress and albumin production [21]. Celi et al [22] evaluated oxidative stress in dairy cows in the transition period and observed lower plasma albumin concentrations near calving in comparison with a higher concentration at 21 days post-calving, indicating that the cows were under oxidative stress.

The HBCS group had a higher urine pH value than the LBCS group pre-calving. Alterations in urinary pH were associated with increased urinary production of Ca, and the metabolic acidosis could have increased the Ca reabsorption of bones and intestines; the Ca absorption has been attributed an increase in the synthesis of 1,25 (OH) 2 D3 [18]. Nevertheless, we did not observe differences in Ca excretion in the urine between the BCS groups (Table 6).

Milk yield was expected to change with the evaluated BCS. However, no differences were detected in milk yield between both BCS groups, although the HBCS group produced 0.75 kg more. Experiments with dairy cows [5,8,23] have shown that animals with a high BCS at calving had a higher milk fat content. This result may be related to the greater mobilization of body reserves in animals with a higher BCS due to the greater mobilization of body fat (non-esterified fatty acid) from the adipose tissue into the bloodstream [23]. This change can contribute to increasing the group of fatty acids that form the milk fat, thus favoring the capture of long-chain fatty acids from the blood to the mammary gland and resulting in greater incorporation of the milk [23]. Anitha et al [7] evaluated a BCS classification system in Murrah buffaloes in different BCS groups and reported that BCS at calving influenced the milk composition (group 1: 2.5 to 2.99; group 2: 3.0 to 3.49; group 3: 3.5 to 3.99; and group 4: 4.0 to 4.49, which had milk fat contents of 5.82%, 6.80%, 7.76%, and 8.46%, respectively). Mushtaq et al [24] evaluated a system for the classification of BCS in Nili-Ravi buffaloes and the group with an average BCS of 3.0 presented a milk fat content of 4.56%.

The HBCS group showed a higher MUN content (p = 0.001) than LBCS did. Several factors can change the MUN content, especially nutrition, with an increase in protein intake or an increase in ruminal-degraded protein [25]. The body protein from catabolism and the deamination of the excess dietary protein can contribute to the pool of blood urea nitrogen. As the blood is secreted from the mammary gland, urea is diffused into and out of the gland, coming into balance with the blood urea. This process allows MUN to be an excellent predictor of the blood urea and urinary N [26]. The HBCS group showed higher concentrations of erythrocytes and hematocrit post-calving and greater concentrations of MCHC and hemoglobin pre-calving (Table 4). Physiologically, at the end of pregnancy, the number of red blood cells increases as a result of the erythropoietic effect of the chorionic placental somatotropin, progesterone, and prolactin [18]. The erythropoietic effect is the formation of red blood cells, white blood cells (lymphocytes, monocytes, eosinophils, granulocytes, neutrophils, and basophils) and platelets. Similarly, the increased blood volume is a response to the placental uterine circulation and to fetal development, maintaining the tissue oxygenation and blood pressure at adequate levels. However, the nutritional condition can alter the blood volume, the erythropoietic effect, and milk yield. High-producing animals exhibit lower concentrations of blood erythrocytes [27].

The HBCS group showed lower concentrations of leuko cytes during the pre- and post-calving periods and a lower level of segmented neutrophils post-calving. Leukocytes participate in the protection of the host against the pathogen and in the monitoring and removal of non-self antigens. The increased blood leukocyte concentrations can be attributed to the lower nutritional condition of the LBCS group at calving. This occurs because an efficient immune response is based on the interaction and on the balance between different types of cells and their products. As the calving date approaches, the total number of leukocytes increases, mainly as a result of the absolute increase in number of neutrophils [28].

High neutrophil levels were shown by LBCS group pre- (p = 0.032) and post-calving (p = 0.034). The decline in nutritional status and increase in oxidative stress can increase the neutrophil concentration, which is explained by the fact that the phagocytosis of the microorganisms is the main function of neutrophils [28]. This represents one of the main lines of defense of the host against pathogens; leukocytes, mainly, are often produced on a large scale in hosts with bacterial load.

The concentrations of urinary metabolites were similar between both groups in the present experiment, except for Cl (mg/dL), which differed between the groups post-calving. The K and Cl concentrations are necessary to maintain the osmotic pressure and acid-base regulation [29]. In the peripartum, there is a positive correlation between the concentrations of calcium, phosphorus, and albumin in the blood [30]. The low concentrations of calcium and phosphorus in the blood observed in the negative energy balance are possibly necessary to sustain milk production [31]. Fiore et al [32] found differences in the blood concentrations of K, Cl, and Ca between the pre- and post-calving periods.

## CONCLUSION

Buffaloes during the transition period showed some variations in oxidative status related to their metabolic status. After calving, the buffaloes that had high BCS and lipid mobilization showed a more pronounced change in their oxidative status, but improved production performance. These conditions can make buffaloes less sensitive to oxidative stress.

## Figures and Tables

**Table 1:** Ingredients proportion and chemical-bromatological composition of concentrate

Item	Diet
Ingredients (% of DM)	
Grass (Penissetum purpureum)1)	80.0
Ground corn	11.0
Soybean meal	3.60
Cottonseed	3.60
Urea	0.8
Limestone	0.4
Mineral2)	0.6
Chemical composition (% DM)	
Dry matter	38.1
Crude protein	11.4
Neutral detergent fiber	65.3
Non-fiber carbohydrates3)	12.5
Ether extract	2.9

DM, dry matter; CP, crude protein; NDF, neutral detergent fiber; NFC, non-fiber carbohydrates.

1) Nutrient composition (g/kg DM): 250.1 DM, 71.0 CP, 768.2 NDF, and 115.1 NFC.

2) kg of the product contains: 190 g of Ca, 60 g of P, 20 g of S, 20 g of Mg, 35 g of K, 70 g of Na, 15 mg of Co, 700 mg of Fe, 10 mg of Cr, 700 mg of Cu, 40 mg of I, 1,600 mg of Mn, 19 g of Se, 2,500 mg of Zn, 200,000 IU of Vit A, 50,000 IU of Vit D_3_, 1,500 IU of Vit E.

3) Estimated according to Hall [33].

**Table 2:** Body weight, body condition score and clinical parameters in the prepartum and postpartum periods for the groups of body condition score at calving (mean±standard error of the mean)

Item	Group1)		p-value2)		
	
	HBCSC	LBCSC	Group	Week	Inter
BW (kg)					
Prepartum	676.5±17.8	571.5±19.8	<0.001	<0.001	0.003
Postpartum	611.3 ± 14.6	520.0±19.0	<0.001	0.457	0.956
CBW (kg)					
Prepartum	−4.50±2.32	−6.30±6.03	0.352	<0.001	0.050
Postpartum	2.52±1.15	1.84±1.42	0.777	0.588	0.994
BCS (unity)					
Prepartum	4.28±0.06	3.63±0.08	<0.001	<0.001	<0.001
Postpartum	3.50±0.08	2.99±0.11	<0.001	0.696	0.966
CBCS (unity)					
Prepartum	−0.050±0.04	−0.120±0.05	0.346	0.155	0.052
Postpartum	−0.015±0.01	−0.017±0.01	0.703	0.740	0.032
RT (°C)					
Prepartum	38.6±0.09	38.4±0.10	0.191	0.246	0.285
Postpartum	38.2±0.06	38.2±0.06	0.278	0.190	0.156
pH, urine					
Prepartum	7.76±0.03	7.87±0.03	0.033	0.368	0.746
Postpartum	7.33±0.09	7.37±0.13	0.795	<0.001	0.346
HR (beat/min)					
Prepartum	77.0±3.39	75.7±4.16	0.616	0.173	0.898
Postpartum	68.8±1.12	71.7±1.41	0.184	0.029	0.811

BW, body weight; CBW, change in body weight; BCS, body condition score; CBCS, change in body condition score; RT, rectal temperature; HR, heart rate.

1) HBCSC, high body score condition at calving (mean of the group = 4.21); LBCSC, low body condition score at calving (mean of the group = 2.98).

2) Probability values for group, week, and interaction between group×week (Inter).

**Table 3:** Milk yield and composition in buffaloes according to groups of body condition score at calving (mean± standard error of the mean)

Item	Group1)		p-value2)		
	
	HBCSC	LBCSC	Group	Week	Inter
Production (kg/d)					
Milk production	7.64±0.34	6.80±0.41	0.491	0.809	0.974
FCM (4.0%)3)	11.62±0.66	9.84±0.55	0.051	0.165	0.287
Fat	0.500±0.02	0.423±0.03	0.027	0.310	0.327
Protein	0.371±0.01	0.317±0.01	0.100	0.737	0.314
Lactose	0.373±0.02	0.342±0.02	0.404	0.157	0.335
TDE	1.23±0.08	1.05±0.08	0.173	0.466	0.928
NFDE	0.852±0.06	0.728±0.06	0.296	0.569	0.980
Composition (g/100 g)					
Fat	6.43±0.22	5.70±0.28	0.007	0.755	0.095
Protein	4.80±0.14	4.85±0.16	0.899	<0.001	0.504
Lactose	4.71±0.07	4.79±0.09	0.374	<0.001	0.473
TDE	15.39±0.19	15.21±0.22	0.497	0.166	0.182
NFDE	10.58±0.09	10.71±0.10	0.404	0.711	0.213
Casein (%)	3.89±0.12	3.92±0.14	0.925	0.012	0.113
Casein (% da CP)	80.93±0.38	80.42±0.44	0.374	<0.001	0.367
MUN (mg/dL)	28.7±1.68	21.2±1.97	<0.001	<0.001	0.899
SCC (unit/mL)	344.7±105	347.3±135	0.941	0.536	0.900

FCM, fat-corrected milk; TDE, total dry extract; NFDE, non-fat dry extract; CP, crude protein; MUN, milk urea nitrogen; SCC, somatic cell count.

1) HBCSC, high body score condition at calving (mean of the group = 4.21); LBCSC, low body condition score at calving (mean of the group = 2.98).

2) Probability values for group, week, and interaction between group×week (Inter).

3) Fat-corrected milk = (((fat − 40)+(protein − 31))×0.01155+1)×prod; where “Fat” is the fat content (g/kg), “Protein” is the protein content (g/kg) and “Prod” is milk production (kg).

**Table 4:** Weekly means values of hemogram for the different groups of body condition score at calving (mean±standard error of the mean)

Item	Group1)		p-value2)		
	
	HBCSC	LBCSC	Group	Week	Inter
Erythrocytes					
Prepartum	8.34±0.50	6.67±0.57	0.531	0.540	0.112
Postpartum	6.47±1.12	5.86±0.47	<0.001	0.001	0.630
Hematocrit					
Prepartum	34.9±1.05	34.3±0.93	0.277	0.393	0.958
Postpartum	33.9±0.72	32.4±0.96	0.012	0.016	0.476
MCHC					
Prepartum	34.8±0.62	33.8±0.36	0.002	0.323	0.894
Postpartum	32.3±0.96	34.7±1.26	0.053	0.947	0.893
Hemoglobin					
Prepartum	6.47±0.33	5.86±0.34	0.004	0.001	0.636
Postpartum	13.1±1.18	11.1±1.59	0.204	0.318	0.470
MCV					
Prepartum	47.0±2.82	54.1±1.61	<0.001	0.189	0.022
Postpartum	50.4 ±0.99	52.5±1.23	<0.001	0.771	0.970
Leukocyte					
Prepartum	9,181.8±1,179	11,167.4±705	0.001	0.029	0.192
Postpartum	10,562.7±841	11,152.1±1,084	0.016	0.036	0.319
Segmented neutrophil					
Prepartum	4,703.9±763	4,834.2±396	0.336	0.977	0.939
Postpartum	5,008.9±570	5,466.4±732	0.034	0.287	0.259
Red blood cell					
Prepartum	3.85±0.50	4.24±0.57	0.562	0.789	0.767
Postpartum	8.34±1.12	6.67±0.47	0.535	0.536	0.113
Lymphocytes (%)					
Prepartum	38.9±5.40	42.3±2.83	0.305	0.049	0.976
Postpartum	32.2±2.59	36.9±3.13	0.847	0.321	0.193
Neutrophils (%)					
Prepartum	4,629±1,200	4,870±1,160	0.032	0.976	0.947
Postpartum	5,008±2,444	5,466±1,298	0.034	0.287	0.259

MCHC, mean corpuscular hemoglobin concentration; MCV, mean corpuscular volume.

1) HBCSC, high body score condition at calving (mean of the group = 4.21); LBCSC, low body condition score at calving (mean of the group = 2.98).

2) Probability values for group, week, and interaction between group×week (Inter).

**Table 5:** Weekly mean values of blood metabolites of different groups of body condition score at calving (mean ±standard error of the mean)

Item	Group1)		p-value2)		
	
	HBCSC	LBCSC	Group	Week	Inter
Glucose (mg/dL)					
Prepartum	65.4±3.77	73.1±2.79	0.087	0.275	0.659
Postpartum	74.3±2.61	73.6±3.22	0.183	0.040	0.336
Total protein (mg/dL)					
Prepartum	8.42±0.27	7.35±0.19	0.035	0.351	0.517
Postpartum	8.51±0.21	8.13±0.27	0.366	0.552	0.854
Albumin (mg/dL)					
Prepartum	3.02±0.10	2.88±0.07	0.036	0.297	0.225
Postpartum	3.07±0.05	2.99±0.06	0.111	0.298	0.473
Globulin (mg/dL)					
Prepartum	5.33±0.23	4.51±0.17	0.046	0.303	0.525
Postpartum	5.41±0.19	4.98±0.24	0.315	0.415	0.715
Urea (mg/dL)					
Prepartum	19.5±2.58	23.6±1.74	0.327	0.635	0.804
Postpartum	44.3±3.77	38.5±4.64	0.022	0.005	0.278
Total cholesterol (mg/dL)					
Prepartum	20.3±1.09	21.0±0.68	0.156	0.601	0.417
Postpartum	26.8±3.01	34.5±3.69	0.029	0.004	0.280
Triglycerides (mg/dL)					
Prepartum	32.7±7.45	35.5±4.91	0.245	0.126	0.989
Postpartum	41.0±3.43	41.7±4.23	0.618	0.997	0.320
Calcium (mg/dL)					
Prepartum	10.3±0.77	10.2±0.50	0.684	0.172	0.718
Postpartum	7.23±0.62	9.76±0.76	0.003	0.532	0.793
Phosphorus (mg/dL)					
Prepartum	8.27±1.17	8.25±0.77	0.427	0.131	0.267
Postpartum	7.93±0.30	7.74±0.37	0.125	0.250	0.290

1) HBCSC, high body score condition at calving (mean of the group = 4.21); LBCSC, low body condition score at calving (mean of the group = 2.98).

2) Probability values for group, week, and interaction between group×week (Inter).

**Table 6:** Urine metabolites concentrations during the prepartum and postpartum periods (mean±standard error of the mean)

Item	Group1)		p-value2)		
	
	HBCSC	LBCSC	Group	Week	Inter
K (mg/dL)					
Prepartum	159.5±23.4	150.3±25.8	0.940	0.880	0.840
Postpartum	135.0±22.3	150.2±24.9	0.090	0.190	0.030
Cl (mg/dL)					
Prepartum	89.7±10.2	96.8±10.1	0.490	0.100	0.350
Postpartum	58.7±6.88	83.8±8.73	<0.001	0.150	0.510
S (mg/dL)					
Prepartum	8.56±2.10	10.18±2.14	0.630	0.350	0.980
Postpartum	12.3±2.50	12.7±2.78	0.890	0.280	0.590
Ca (mg/dL)					
Prepartum	7.47±1.15	6.03±1.13	0.360	0.280	0.130
Postpartum	12.44±1.40	13.44±1.72	0.840	0.630	0.790
Urea (mg/dL)					
Prepartum	176.4±33.7	260.8±34.2	0.170	0.007	0.050
Postpartum	1,162.4±112	1,204.4±141	0.941	0.014	0.714
Uric acid (mg/dL)					
Prepartum	3.85±0.50	4.22±0.57	0.562	0.787	0.767
Postpartum	12.3±2.50	12.7±2.78	0.899	0.286	0.592

1) HBCSC, high body score condition at calving (mean of the group = 4.21); LBCSC, low body condition score at calving (mean of the group = 2.98).

2) Probability values for group, week, and interaction between group×week (Inter).
